# A Meta-Analysis of Maternal Smoking during Pregnancy and Autism Spectrum Disorder Risk in Offspring

**DOI:** 10.3390/ijerph120910418

**Published:** 2015-08-26

**Authors:** Shiming Tang, Ying Wang, Xuan Gong, Gaohua Wang

**Affiliations:** Mental Health Center, Renmin Hospital of Wuhan University, Jiefang Road 238#, Wuhan 430060, China; E-Mails: tsm8845@163.com (S.T.); wangying@whu.edu.cn (Y.W.); gongxuan1976@163.com (X.G.)

**Keywords:** maternal smoking, risk factor, autism spectrum disorder, meta-analysis

## Abstract

The association between maternal smoking during pregnancy and autism spectrum disorder (ASD) risk in offspring has been investigated in several studies, but the evidence is not conclusive. We, therefore, conducted this meta-analysis to explore whether an association exists between maternal smoking during pregnancy and ASD risk in offspring. We searched PubMed, Embase, Web of Science, and the Cochrane Library for studies of maternal smoking during pregnancy and ASD risk in offspring up to 10 June 2015. The random-effects model was used to combine results from individual studies. 15 observational studies (6 cohort studies and 9 case-control studies), with 17,890 ASD cases and 1,810,258 participants were included for analysis. The pooled odds ratio (OR) was 1.02 (95% confidence interval (CI): 0.93–1.13) comparing mothers who smoked during pregnancy with those who did not. Subgroup and sensitivity analysis suggested the overall result of this analysis was robust. Results from this meta-analysis indicate that maternal smoking during pregnancy is not associated with ASD risk in offspring. Further well-designed cohort studies are needed to confirm the present findings.

## 1. Introduction

Autism Spectrum Disorders (ASDs) are a group of heterogeneous neurodevelopmental conditions characterized by social and communication difficulties, and by restricted and repetitive interests and behaviors [1]. The ASDs include autistic disorder, Asperger syndrome, atypical autism, and other or unspecified pervasive developmental disorders [2]. The global prevalence of ASDs is estimated to be 1 person in 132, accounting for more than 7.7 million disability-adjusted life years worldwide in 2010 [3]. Although the cause of ASDs still remains unknown, they are regarded as multifactorial, with many risk factors acting together to produce the phenotype [4]. The identification of modifiable environmental risk factors is of great importance for the primary prevention of ASDs.

Smoking during pregnancy may lead to numerous adverse outcomes in the offspring, such as low birth weight, placenta abruption, and sudden infant death syndrome [5]. Maternal smoking is also a risk factor for several neurodevelopmental disorders, including attention-deficit, conduct disorder, and antisocial behavior [6]. Several studies have explored the relationship of maternal smoking during pregnancy and ASDs risk, but the results were inconclusive. A recent meta-analysis by Roson *et al.* concluded that there was no association between maternal prenatal smoking and ASD in offspring based on 15 observational studies [7]. However, Roson *et al.* [7] only searched two databases which missed several eligible studies. In addition, Rosen *et al.* misunderstood the difference between the period of “prenatal” and “pregnancy”, and they inappropriately included studies that only reported smoking during pregnancy but not specified whether it was during the prenatal period or not. We therefore performed this study to systematically assess the association of maternal smoking during pregnancy (including both prenatal and perinatal smoking) and ASDs risk.

## 2. Methods

### 2.1. Data Sources and Searches

This meta-analysis was performed according to the Meta-Analysis of Observational Studies in Epidemiology guidelines [8]. A systematic search of PubMed, EMBASE, Web of Science, and Cochrane Library up to 10 June 2015 was conducted to identify relevant studies regarding the association between maternal smoking during pregnancy and ASDs risk. A reference list of the related studies were further screened for any additional literatures. The search terms for PubMed were: (smoke OR smoking OR tobacco OR risk factors) AND (autism spectrum disorder or autism) AND (pregnancy OR prenatal OR perinatal OR maternal). Similar search strategies were used in other databases. We applied no language restriction in the process of literature search and selection.

### 2.2. Study Selection

Studies were included if they met the following criteria: (i) had a cohort or case-control design; (ii) reported the association between maternal smoking during pregnancy (including prenatal or perinatal smoking) and ASD risk in their offspring; and (iii) reported risk estimates and 95% confidence interval (CI). Only the study with the most complete data was included if data was reported more than once in different literatures. Non-human studies, letters, case reports, conference abstract, review, and studies that with insufficient information were excluded.

### 2.3. Data Extraction and Quality Assessment

Two reviewers independently extracted data, any disagreements were resolved by discussion. The following data were extracted: last name of the first author, publication year, study design, study location, age, sex, number of cases and total population, risk estimates adjusted for the maximum number of confounding variables with corresponding 95% CIs, and confounding factors that were being adjusted for in the analysis. We also extracted any dose-relationship data for smoking and ASDs risk.

The methodological quality was assessed using the Newcastle-Ottawa Scale (NOS) [9]. The NOS has been widely used for quality assessment of cohort and case-control studies, a maximum of nine scores were assigned to eight items which indicated the methodological quality of each study. We divided the study quality into three categories (1) high quality (scored 7–9); (2) moderate quality (scored 4–6); and (3) low quality (scored 0–3).

### 2.4. Data Analysis

We combined the results using the OR as a measure of the association between smoking and ASD risk. Heterogeneity was investigated by the Cochrane Q statistic (significance level at *p* < 0.10) and the *I*^2^ statistic [10,11]. The random effect model was used to calculate the pooled OR if *p* > 0.10 and *I*^2^ ≤ 50%, otherwise the fixed effect model was chosen [12]. Sensitivity analysis was performed to assess whether any individual study significantly affected pooled estimates by omitting one study in each turn. Additionally, subgroup analyses were performed according to study design, study location, and whether they were adjusted for confounding variables to examine the source of potential heterogeneity. We used both Begg’s test [13] and Egger’s test [14] to detect publication bias. Stata version 11.0 (Stata Corporation) was used for all analysis. All statistical tests in this study were two-sided with a significance level of 0.05, unless otherwise specified.

## 3. Results

### 3.1. Literature Search and Selection

**Figure 1:**
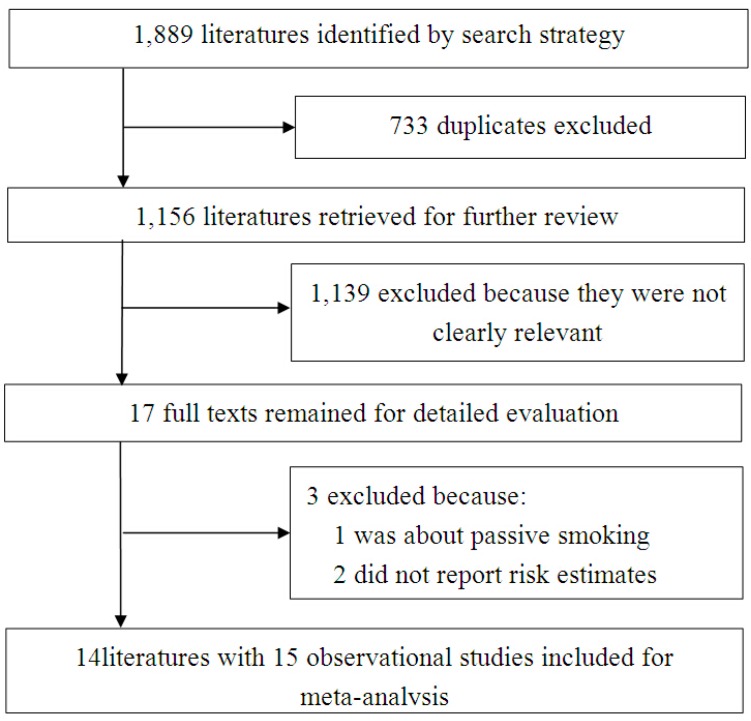
Flow diagram of the study selection process.

Figure 1 shows a flow diagram of the selection process. A total of 1889 literatures were identified by the search strategy. After removal of duplicate literatures, 1156 articles were left for screening. By screening the titles and abstracts, 1140 articles were excluded as they were not clearly relevant. After detailed evaluation of the 16 full texts, one article [15] that reported passive smoking and two articles [16,17] that did not reported risk estimates was excluded. Due to the fact that one article [18] met the inclusion criteria reported results based on two separate cohort studies, 14 articles [6,18,19,20,21,22,23,24,25,26,27,28,29,30] with 15 studies were included for meta-analysis.

### 3.2. Study Characteristics

Table 1 listed the characteristics of the included studies. Of the 15 include studies, there were six cohort studies [18,20,21,26,30] and nine case-control studies [6,19,22,23,24,25,27,28,29], with a total of 17,890 cases and 1,810,258 participants involved. 10 studies [6,18,22,23,25,26,27,28,29] were from Europe (Denmark, Sweden, Finland, Norway and Poland) while five studies [19,20,21,24,30] were from America (Canada and US). The information about smoking was generally collected during pregnancy, and ASDs were ascertained by medical records in most of the included studies. Overall, the methodological quality was good (average score: 7.3), with 12 studies [6,18,19,21,22,23,24,25,27,28,30] in high quality and three studies [20,26,29] in moderate quality (Supplemental Table 1).

**Table 1:** Characteristics of included studies of maternal smoking during pregnancy and ASD risk in the meta-analysis.

Study	Location	Design ^a^	ASD Cases/Total Participants	Age(y), Sex	Ascertainment of Smoking	Ascertainment of ASDs	OR(95%CI)	Confounding
Hultman 2002 [23]	Sweden	CC	408/2448	<10, M&F ^b^	Collected by midwives at registration for antenatal care	Medical record	1.4(1.1,1.8)	Maternal age, parity, mothers’ country of birth, hypertensive diseases, diabetes, mode of delivery, pregnancy bleeding, season of birth, gestational age, birth weight for gestational age, Apgar at 5 min, congenital malformations
Larsson 2005 [25]	Denmark	CC	249/6474	≤24, M&F	Reported at the first antenatal visit	Medical record	1.06(0.80,1.39)	NA
Maimburg 2006 [28]	Denmark	CC	473/5203	<10, M&F	Collected by midwives at the first antenatal visit	Medical record	0.9(0.7,1.4)	Mother and fathers age, mothers citizenship, birth weight and gestational age, Apgar, birth defect and irregular foetal position
Bilder 2009 [19]	US	CC	132/13,332	8, M&F	NA^c^	Clinician review of medical and school record	0.506(0.222,1.152)	NA
Larsson 2009 [26]	Sweden	C	72/4779	6–8, M&F	Parent-report collected for pregnancy when child was age 1–3 years	Parent-report collected by follow-up questionnaire	2.09(1.08,4.03)	Unadjusted
Burstyn 2010 [20]	Canada	C	1138/218,890	≤9, M&F	Collected on admission to hospital for delivery	Medical registry record	0.86(0.72,1.02)	Maternal age, maternal weight, maternal height, pre-pregnancy diabetes, gestational diabetes, bleeding, weight gain, parity, socio-economic status, pre-eclampsia, presentation, type of labour, delivery by caesarian section, gestational age, birth weight, apgar at 1 min, Apgar at 5 min, birth year
Dodds 2010 [21]	Canada	C	924/129,733	1–17, M&F	Investigated by a standardized questionnaire	Administrative databases with relevant diagnostic information	0.93(0.81,1.08)	Unadjusted
Haglund 2011 [22]	Sweden	CC	157/68,964	8–15, M&F	Swedish Medical Birth Registry	Medical registry	0.7(0.5,1.0)	Year of birth, maternal age at delivery, parity , sex, gestational age at birth, standard deviations scores, obstetrical risk factor
Kalkbrenner 2012 [24]	US	CC	3315/633,989	8, M&F	Birth certificate data	Surveillance-ascertained	0.9(0.8,1.01)	Maternal education, race and ethnicity, marital status, maternal age, county population size, birth year and surveillance site.
Lee 2012 [27]	Sweden	CC	3958/42,941	4–17, M&F	Recorded by midwives at the first prenatal visit	Medical registry	1.10(1.01,1.20)	Unadjusted
Tran 2013 [6]	Finland	CC	4019/20,601	≤7, M&F	Collected by maternity clinic nurses during routine obstetric visits	Medical registry	1.0(0.9,1.2)	Maternal age, maternal mental diagnosis, socioeconomic status and weight for gestational age
Nilsen 2013 [18]	Norway	C	2072/507,856	3–11, M&F	Recorded by check boxes at the beginning and the end of pregnancy	Medical registry	1.20(0.84,1.71)	Year of birth, maternal age, marital status, hospital size.
	Norway	C	234/89,836	3–11, M&F	The same as above	Medical registry	1.17(1.04,1.31)	The same as above
Mrozek-Budzyn 2013 [29]	Poland	CC	96/288	2–15, M&F	Investigated by a standardized questionnaire	Medical record	3.32(1.23,9.82)	Unadjusted
Xiang 2015 [30]	US	C	643/64,924	1.5–2 M&F	Extracted from medical records and birth certificate records	Screened by a modified version of CHAT ^d^ and diagnosed by pediatric developmental specialist evaluation	0.83(0.33,2.09)	Birth year

^a^ C: cohort study CC: case-control study; ^b^ NA: not available; ^c^ M&F: male and female; ^d^ CHAT: Checklist for Autism in Toddlers.

### 3.3. Main Analysis

**Figure 2:**
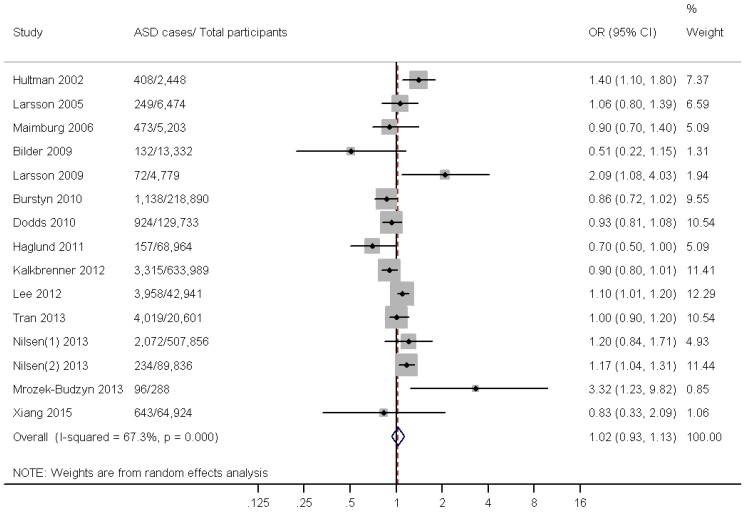
A forest plot of the association between maternal smoking during pregnancy and ASD risk.

The meta-analysis data that explored the effect of maternal smoking during pregnancy on the risk of ASDs is shown in Figure 2. A statistically significant heterogeneity was found across studies (*p*_heterogeneity_ < 0.01, *I*^2^ = 67.3%). The pooled OR estimates based on 15 studies showed that maternal smoking during pregnancy was not associated with an increased risk of ASDs (OR = 1.02; 95% CI: 0.93–1.13).

### 3.4. Subgroup and Sensitivity Analysis

Table 2 presents the results of subgroup analysis stratified by study design, study location, and adjustment for confounders. Maternal smoking during pregnancy was associated with significantly reduced risk of ASDs in the North American populations (OR = 0.90, 95%CI: 0.83–0.97), but not European populations (OR = 1.11, 95%CI: 0.99–1.24). In subgroup analysis by study design and adjustment for confounders, the difference in pooled ORs across strata was not statistically significant. For the sensitivity analysis, the pooled ORs did not vary materially ranging from 1.00(95% CI: 0.91–1.10) when omitted Hultman *et al.* study [23] to 1.05(95% CI: 0.95–1.15) when excluded Dodds *et al.* study [21].

**Table 2:** Summary results of association between maternal smoking during pregnancy and ASD risk.

Variables	No. of Studies	*I*^2^%	*p_heterogeneity_*	OR(95%CI)
All studies	15	67.3	<0.001	1.02(0.93,1.13)
Location				
Europe	10	60.1	0.007	1.11(0.99,1.24)
America	5	0.00	0.671	0.90(0.83,0.97)
Design				
Cohort	6	68.9	0.007	1.05(0.88,1.25)
Case-control	9	70.0	0.001	1.01(0.88,1.15)
Study quality				
High	12	60.4	0.003	1.02(0.92, 1.12)
Moderate	3	83.1	0.003	1.65(0.73, 3.73)
Adjustment for confounders				
Yes	9	69.0	0.001	1.00(0.88,1.13)
No/NA ^*^	6	68.2	0.008	1.08(0.89,1.32)

^*^ NA: not available.

### 3.5. Publication Bias

Begg’s test and Egger’s test suggested no significant publication bias among the included studies (all *p* > 0.05).

## 4. Discussion

The present study used a comprehensive search strategy and explored the relationship between maternal smoking during pregnancy and ASDs based on 15 observational studies. However, the overall results did not suggest a significant association between maternal smoking during pregnancy and ASDs risk.

Several hypotheses of association between maternal smoking during pregnancy and ASD are plausible. First, nicotine is the chemical that most probably has adverse effects on brain development among thousands of ingredients of tobacco smoke [31] and its effects are thought to occur via its action on nicotinic acetylcholine receptors [32]. Second, women with lower socioeconomic status (SES) have a higher tendency to smoke during pregnancy compared with those with higher SES [33,34]; nevertheless, low parental SES is associated with an increased risk of ASD [35]. A third explanation is the transmission of genetic risk from mothers to their offspring, as ASD is highly genetic [4] and smoking during pregnancy is more likely to happen in women with a predisposition to mental health and behavioral problems [36]. Another hypothesis was that maternal smoking related high levels of intrauterine testosterone might contribute to the increased ASD risk [37].

Despite all the hypotheses mentioned above, the pooled OR failed to find a positive association between maternal smoking during pregnancy and ASDs. However, in the subgroup analysis, a significant negative association was observed in the North America. Although the association was not statistically significant in the individual studies of this subgroup, the increased sample size narrowed the confidence interval of the pooled result and thus it became statistically significant. Nevertheless, further studies are needed to examine this contrary finding, considering that the results were not fully adjusted and potential bias may exist due to the design of included studies.

Our study has several limitations that deserve consideration. First, because this meta-analysis was based on observational studies, our results were prone to recall and selection bias inherent in the included studies. In addition, the potential confounding from other risk factors could not be ruled out, especially when considering that some studies only reported crude risk estimated without adjustments. Second, potential misclassification of maternal smoking may lead to incorrect estimates of associations. The smoking status was generally collected at early pregnancy and was based on self-report, there was a possibility that participants tend to narrow their smoking status and the smoking habit might change during pregnancy. Third, high statistical heterogeneity was found across the studies that may weaken the strength of our findings; however, the subgroup analysis, as well as sensitivity analysis, showed that results from our meta-analysis was robust. Finally, the included studies of our meta-analysis were mainly conducted in the North Europe and the North America. The findings of this study had limited implications for other populations, like the Africans and Asians, as both the genetic and environmental factors in relation to autism varied among different ethnicities.

## 5. Conclusions

This meta-analysis indicates that there is no association between maternal smoking during pregnancy and ASD. Given the limited number of cohort studies included in this study, the present findings should be confirmed in further prospective cohort studies.

## Supplemental Material

**Supplemental Table 1:** Quality assessment based on the Ottawa-Newcastle Scale.

Case-Control Study	Definition of Cases	Representativeness of the Cases	Selection of Controls	Definition of Controls	Comparability of Cases and Control ^*^	Ascertainment of Exposure	Same Method of Ascertainment for Cases and Controls	Non-Response Rate	Total Score
Hultman 2002	1	1	1	1	2	1	1	0	8
Larsson 2005	1	1	1	1	1	1	1	0	7
Maimburg 2006	1	1	1	1	2	1	1	1	9
Bilder 2009	1	1	1	1	1	0	1	1	7
Haglund 2011	1	1	1	1	1	1	1	1	8
Kalkbrenner 2012	1	1	1	0	1	0	1	1	6
Lee 2012	1	1	1	0	2	1	1	0	7
Tran 2013	1	1	1	1	2	1	1	1	9
Mrozek-Budzyn 2013	1	1	0	0	2	1	1	0	6
Cohor study	Representativeness of the exposed cohort	Selection of the non exposed cohort	Ascertainment of exposure	outcome of interest was not present at start of study	Comparability of cohorts *	Assessment of outcome	follow-up long enough for outcomes to occur	Adequacy of follow up of cohorts	
Larsson 2009	1	1	0	0	0	1	1	1	5
Burstyn 2010	1	1	1	0	1	1	1	1	7
Dodds 2010	1	1	1	1	2	1	1	1	9
Nilsen(1) 2013	1	0	1	1	1	1	1	1	7
Nilsen(2) 2013	1	0	1	1	1	1	1	1	7
Xiang 2015	1	1	1	1	0	1	1	1	7

**^*^** A maximum of 2 points were assigned to this item.
